# Knowledge-augmented pre-trained language models for biomedical relation extraction

**DOI:** 10.1186/s12859-025-06262-6

**Published:** 2025-10-07

**Authors:** Mario Sänger, Ulf Leser

**Affiliations:** 1 Department of Computer Science, Humboldt-Universität zu Berlin, Unter den Linden 6, 10099 Berlin, Germany; 2 Enterprise AI Platforms & Services, AstraZeneca, Av. Diagonal, 615, 08028 Barcelona, Spain

**Keywords:** Biomedical natural language processing, relation extraction, Pre-trained language models, Knowledge augmentation, Benchmark

## Abstract

Automatic relationship extraction (RE) from biomedical literature is critical for managing the vast amount of scientific knowledge produced each year. In recent years, utilizing pre-trained language models (PLMs) has become the prevalent approach in RE. Several studies report improved performance when incorporating additional context information while fine-tuning PLMs for RE. However, variations in the PLMs applied, the databases used for augmentation, hyper-parameter optimization, and evaluation methods complicate direct comparisons between studies and raise questions about the generalizability of these findings. Our study addresses this research gap by evaluating PLMs enhanced with contextual information on five datasets spanning four relation scenarios within a consistent evaluation framework. We evaluate three baseline PLMs and first conduct extensive hyperparameter optimization. After selecting the top-performing model, we enhance it with additional data, including textual entity descriptions, relational information from knowledge graphs, and molecular structure encodings. Our findings illustrate the importance of (1) the choice of the underlying language model and (2) a comprehensive hyperparameter optimization for achieving strong extraction performance. Although inclusion of context information yield only minor overall improvements, an ablation study reveals substantial benefits for smaller PLMs when such external data was included during fine-tuning.

## Introduction

Automatic relationship extraction (RE) from biomedical literature is an essential tool for managing and synthesizing the ever-increasing volume of scientific knowledge produced annually. This technology enables researchers to identify and analyze large sets of complex interactions between genes, diseases, drugs, and other biomedical entities within the literature, significantly accelerating the pace of scientific discovery [45]. For instance, in fields such as pharmacovigilance and drug development, accurately identifying potential drug interactions is vital as these interactions can lead to adverse effects or diminished therapeutic efficacy [22]. Accordingly, methods for extracting relationships from biomedical texts have been investigated intensively over the last two decades [51].

In recent years, pre-trained language models (PLMs) have become the prevalent technology in relation extraction [29, 43]. These methods usually leverage a transfer learning setting, i.e., the language model is pre-trained on extensive in-domain text collections first and then fine-tuned to the specific relation extraction task using human-labeled gold standard data sets [30]. For improving the fine-tuning process, several studies explore utilizing additional context information, including curated entity definitions [4], knowledge graph triplets [6, 39], and chemical structure information [5], for improving the fine-tuning of PLM-based relation extraction methods. For example, Aldahdooh et al. [1] combine PLMs with gene descriptions from the Entrez Gene database [36] and chemical descriptions from the Comparative Toxicogenomics Database (CTD) [13] for improved mining drug-target interactions. Dou et al. [16] utilize human-curated descriptions of different drug aspects from DrugBank to enhance a PLM-based baseline model for drug-drug interaction prediction. Furthermore, Asada et al. [6] leverage knowledge graph information to augment a PubMedBERT-based drug-drug interaction identification model. Similarly, Sousa et al. [39] explore utilizing class and relational data from four different knowledge bases for drug-drug, chemical-disease, and phenotype-gene relation extraction using SciBERT [8]. Most of these studies report strong advantages when incorporating additional contextual information when compared to approaches that train the PLMs exclusively on annotated texts [1, 6, 16, 37, 39]. A direct comparison of published results, however, usually is not possible due to technical differences (e.g., choice of the underlying language model, used databases, model design) and applied evaluation procedure. This raises questions about the determining factors for the improvements achieved and the transferability of the studies’ insights to other RE scenarios and to other PLMs. For instance, our investigation in [43] explored knowledge-enhanced PLMs for identifying chemical-protein relations and found that context information achieves only marginal improvements if the hyperparameters of the PLM were carefully optimized. It thus an open research question whether RE based on PLMs actually benefit from additional information or not - and under which circumstances.

In this study we address this research gap by assessing PLMs augmented with different types of additional context information in different relation extraction use cases. We utilize five data sets encompassing four biomedical relation scenarios within a uniform evaluation setting, allowing to derive more reliable insights into the effectiveness of including context information in the fine-tuning process of PLMs. For each data set, we perform an extensive hyperparameter optimization of three baseline PLM models (i.e., PubMedBERT [20], RoBERTa-large-PM-M3-Voc [31], BioLinkBERT-Large [48]) first, before extending the best model with additional data. We explore the inclusion of (a) textual entity descriptions, (b) embedded information representing an entity’s neighborhood in a knowledge graph as well as its mentions in the literature [38], and (c) molecular structure encodings for drug- and chemical-related scenarios.

Our experimental results highlight a superior performance of the BioLinkBERT-large model across all extraction scenarios. However, we mostly achieve no or only minor performance improvements when including additional context information in the fine-tuning procedure compared to adapting the model on gold standard annotations only. To verify this result, that potentially contradicts several previous studies, we perform an ablation study on the sizes of the PLM models used, and reveal that the extraction quality of PLM-based models having fewer parameters benefit considerably from incorporating additional external information while fine-tuning - an effect that vanishes with more recent and considerably larger models. These findings suggest that the larger PLMs implicitly encode (to some extent) the supervision signals from the additional information.

## Material and methods

### Data sets

We investigate our method on five data sets spanning four relationship scenarios, i.e. chemical-disease, chemical-gene, drug-drug, and gene-disease, providing a more comprehensive evaluation than in prior art. We leverage the following datasets:

- Chemical-disease: BioCreative-V-CDR (BC5CDR) [32]
- Chemical-gene: ChemProt [28], CPI [15]
- Drug-drug: DDI corpus [23]
- Gene-disease: ChemDisGene (CDG) [49]

Note, we utilize two data sets for chemical-protein relations to analyze and account for possibly existing data set-specific patterns or biases. Two of the five data sets (BC5CDR, ChemDisGene)) have only document-level annotations of relationships, while the other three come with mention annotation. See Tables 1 and  2 for basic statistics as well as entity and relation types of the used data sets and refer to Appendix 1 for an in-detail description. In order to link the entity mentions to information in knowledge bases, we normalize the entity mentions to shared ontologies, i.e., NCBI Gene [11] for genes, CTD Diseases^1^ [13] for diseases, and CTD Chemicals^2^ [13] for chemicals. For BC5CDR and ChemDisGene, we use the provided gold standard MESH and NCBI Gene annotations. We apply different strategies for the remaining data sets:

- ChemProt: For ChemProt, we utilize the annotations provided by PubTator Central [44] using the PubMed identifiers of the data set.
- CPI: For normalizing protein annotations to NCBI Gene, we leveraged the UniProt identifiers [42] given in the data set and the id-mapping service of the platform.^3^ Chemical mentions are mapped to MESH by applying a cross-reference table^4^ on the given PubChem identifiers [26] of the data set.
- DDI: Since the DDI texts essentially originate from Drugbank, which strongly follows the standard nomenclature, we rely on string matching for mapping the drug mentions to CTD chemicals.

See Table 7 for statistics on the number of normalized mentions and unique entities.

**Table 1:** Overview of basic statistics of the data sets used in our evaluation

Data set	Text type	Instances	Tokens	Splits		
				Train	Val.	Test
BC5CDR (DL)	Abstracts	1.500	281.792	500	500	500
Chemprot (ML)	Abstracts	2.482	563.091	1020	612	800
CPI (ML)	Abstracts^†^	1.808	67.163	1808	0	0
				(1208)	(300)	(300)
ChemDisGene (DL)	Abstracts	523	123.767	523	0	0
				(300)	(80)	(123)
DDI corpus	Abstracts & DrugBank Texts	1.017	167.148	651	0	191
				(550)	(101)	

Moreover, we illustrate whether the corpus provides mention—(ML) or document-level (DL) relation annotations. If a data set, does not provide a validation and/or test split, we give the size of the created split in parenthesis below.

^†^ CPI contains only selected sentences from the abstract and not the entire text

Numbers in parentheses indicate the size of the splits we created when the dataset does not provide them

**Table 2:** Overview of entity types and relation types of the data sets used in our evaluation

Data set	Entities		Relations	
	Type	Count	Type	Count
BC5CDR	Chemical	15.953	Chemical-disease	3.169
	Disease	13.318		
Chemprot	Chemical	32.514	Upregulator	2.061
	Gene	30.922	Downregulator	5.098
			Agonist	497
			Antagonist	741
			Substrate	1.873
CPI	Chemical	3.563	Chemical-protein	2.931
	Gene	4.256		
ChemDisGene	Chemical	5.739	Marker/Mechanism	494
	Disease	2.931	Therapeutic	82
	Gene	5.578		
DDI corpus	Brand	1.865	Advise	1.047
	Drug	12.405	Effect	2.047
	Group	4.221	Interaction	285
			Mechanism	1.621

For each data set we report the number of total entity mentions and unique entities, identified by the given entity normalization identifiers of the data set (BC5CDR and ChemDisGene) or unique surface forms (Chemprot, CPI and DDI)

### Base model

**Fig. 1:**
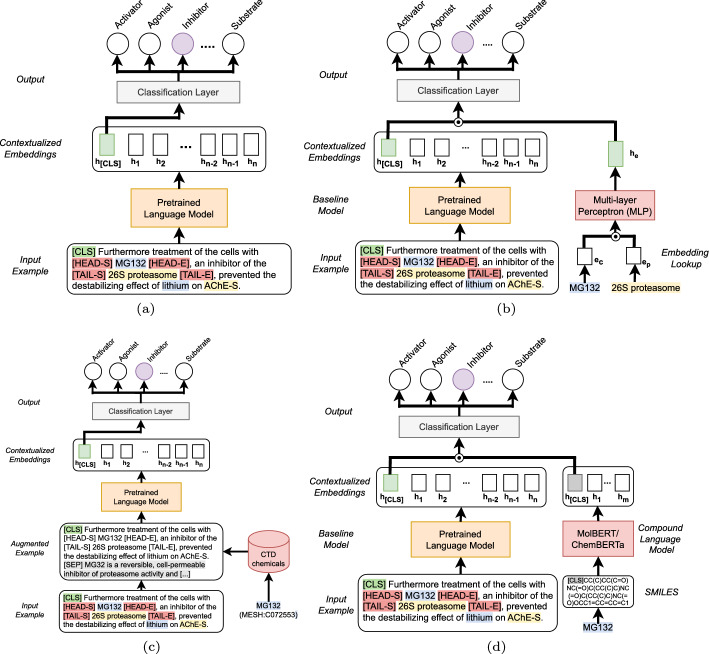
Overview of the baseline model and all extensions we evaluate in our study. **a** Baseline model: We build one input example per entity pair, i.e., chemical-protein pair, in each sentence and mark the pair under investigation with special tokens. The sentence is embedded using a pretrained language model and the [CLS] token embedding is passed through an output layer performing the relation classification. **b** Model enhanced with additional embedded entity information: First, we lookup the pre-trained KB embeddings for the head and tail entity under investigation and input the concatenation of both to a multi-layer perceptron (MLP). The resulting embedding is concatenated to the contextualized input text embedding as additional input to the classification layer. **c** Model augmented with additional textual information: We conduct KB lookups to retrieve textual descriptions for the head entity, the tail entity or both using entity identifiers. We include the textual descriptions by appending them to the input text separated by the [SEP] token. **d** Model extended with molecular information of chemicals: For a given chemical *c*, we first retrieve its respective SMILES string $SM_c$, prepend the [CLS] token to $SM_c$ and feed it into the compound language model. We use the representation of the [CLS] token in the last hidden layer as encoding for the structure of *c* and concatenate it with the language model’s output to form the classification layer’s input

We follow common practice [30] and model relation extraction as a multilabel, sentence-level relation classification problem, which we approach by fine-tuning a pre-trained transformer-based language model. More specifically, we generate one training/testing example per pair of entities that occur together in the same sentence. We mark the entity pair under investigation by inserting the special tokens [HEAD-S], [HEAD-E], [TAIL-S], and [TAIL-E], which highlight the beginning and end of the head and of the tail entity. Moreover, we prepend the [CLS] token, a specially designed token to aggregate the information of the entire input text in PLMs, to each input example. We only form entity pairs that comply with the entity types of the respective relation type. We follow the data set standard for DDI by creating only one input instance per drug-drug pair based on the order of occurrence in the input text. We use a pre-trained language model to obtain a contextualized embedding $h_i$ of every token in the sentence and represent the sentence by using the embedding of the [CLS] token. Finally, we apply a linear layer to the sentence representation and transform the activation score with a sigmoid nonlinearity. See Fig. 1a for an illustration of the baseline model.

We inspect two approaches for enhancing the input text separately. First, we add the sentence before and after the sentence under examination to the input text. This augmentation is intended to provide the model with additional textual context to make more informed relation decisions. Second, we prepend a verbal task instruction to the input text:

*Is there a* <*relation-type*>*interaction between* <*head-entity*>*and* <*tail-entity*>*?*

In the instruction, we replace the placeholders <relation-type>, <head-entity>, and <tail-entity>with the focused relation type and the specific entity mentions of the input example.

### Model extensions

In the following paragraphs, we describe how the base model is augmented with additional entity information aiming to provide contextual knowledge beyond the text under consideration. We believe that the extended context information can offer valuable insights to the model when deciding upon relationships between entities. Depending on the type of the entities investigated different information are added, i.e., textual descriptions and embedded information for all considered entity types as well as molecular structure information for drug-/chemical-related use cases. The inclusion of all three types of additional entity information have been investigated in prior work, e.g., refer to [1, 16] for textual [4, 39], and [35, 41] for molecular information. Yet, there has been no systematic comparison that evaluates all these types within a single, unified setting.

#### Additional textual information

We hypothesize that augmenting the input with additional textual information beyond the sentence context could result in a more accurate model. For instance, in case of chemical-protein relation extraction, this could include information indicating that a chemical is known to function as an antagonist for a particular group of proteins or that a protein is associated with a specific protein family.

We experiment with diverse additional textual entity information gathered from different database bases, i.e., *CTD chemicals* for chemicals/drugs, *CTD diseases* for disease, and *NCBI gene* for genes. For instance, in cases where the chemical *c* and the disease *d* are marked as the head and tail entity, respectively, we conduct database queries to retrieve the textual description of both *c* and *d*. The retrieved information is appended to the input data (see Fig. 1c). We use the [SEP] token to separate the original input and additional text. In cases where this led to a total number of tokens exceeding the maximum sequence length of the PLM, we first truncated the context information before truncating the input sentence. Appendix 4 provides an overview of the used databases and the number of entity mentions for which we could obtain a textual description per data set.

#### Additional entity embeddings

Next to the textual information, we evaluate the inclusion of entity embeddings trained via knowledge base embedding methods, as they are capable of encoding topological information of knowledge bases (KBs) into dense vectors that can be used to infer relations between entities in the KB [3]. For this, we experimented with two methods, MuRE [7] and RotatE [40], trained on graphs representing interactions recorded in the CTD, i.e., chemical-gene,^5^ chemical-disease,^6^ and chemical-phenotype interactions.^7^ We build three knowledge graphs from the data, one having all entity types and interactions included and two exclusively focused on chemical-gene and chemical-disease relations, respectively. Refer to Appendix 5 for statistics of the used knowledge graphs.

Given an input example of our model with chemical *c* and disease *d*, we concatenate the corresponding KBE embeddings $e_c$ and $e_d$ and feed them through a two-layer Multilayer Perceptron. We freeze the knowledge base embeddings while fine-tuning the PLM. The obtained embedding $h_e$ is concatenated with the sentence embedding immediately before the output layer (see Fig. 1b).

In addition to knowledge base embeddings, we explore the inclusion of contextual information from the mentions of the entities in the entire biomedical literature. This contextual information might offer additional insights into their relationships with other biomedical concepts not represented in curated knowledge bases. For this, we leverage dense semantic entity representations given by [38]. Integrating literature entity embeddings is conducted similarly to the approach applied to KB embeddings.

#### Additional structural information

Finally, we assess the impact of incorporating molecular structure information in chemical- and drug-related extraction scenarios. Like textual and embedded entity information, the similarity in drug molecular structures may offer valuable signals for predicting connections with other entities. To capture the molecular characteristics of chemicals, we first retrieve their SMILES representation [46] from DrugBank and then explore three distinct encoding methods: molecular fingerprints, MolBERT [17] and ChemBERTa [12].

Molecular fingerprints represent molecules using fixed-sized binary feature vectors usually based on the presence of common substructures [10] or topological information [9]. We integrate the fingerprints similarly to the KGE embeddings (see Fig. 1b). First, we lookup the pre-computed fingerprint vector $fp_c$ for a given chemical *c*, then input $fp_c$ into a two-layer perceptron and concatenate the network output with the text embedding.

MolBERT [17] and ChemBERTa [12] are transformer-based compound language models trained on large compound repositories, i.e., CHEMBL [19] and ZINC [25], which allow computing dense vector representations of chemicals. We adapt their original implementations^8^ and integrate them into our baseline model as follows: for a given chemical *c* we first retrieve the respective SMILES string $SM_c$, prepend the [CLS] token to $SM_c$ and feed it into the compound model. Next, we use the representation of the [CLS] token in the last hidden layer as encoding for the structure of *c* and concatenate it with the language model’s output to form the classification layer’s input. Figure 1d illustrates the integration of both models.

## Experiments and results

### Evaluation setting

Our study comprehensively evaluates the performance of pre-trained language models for biomedical relation extraction in a unified and consistent setting for four types of relationships: chemical-disease, chemical-gene/protein, drug-drug, and gene-disease interactions. We evaluate our approach in two steps. First, we perform a hyperparameter optimization of our baseline approach using three different PLMs and one fixed random seed (907). Based on these results, we use the three best configurations for each PLM and perform two additional runs using different seeds. We average the results of the three runs per PLM and choose the best setting based on validation set performance.

Next, we examine the inclusion of the additional entity information using the best-performing PLM as the underlying language model. We investigate different configuration options and hyperparameter settings using a fixed seed for each additional information. However, we keep the hyperparameters of the base model fixed. Analogously to the baseline models, we perform two additional runs using different seeds for the best configuration found and report the average performance.

We run the experiments on NVIDIA A100 GPUs having 80 GB main memory. Each experiment is executed on a single GPU taking between 10 min and two hours to finish depending on the configuration (e.g., data set and batch size used) applied.

#### Base models

Our base models are built on three pre-trained language models: PubMedBERT [20], RoBERTa-large-PM-M3-Voc [31] and BioLink-BERT-Large [48]. The latter two models are considerably larger in terms of parameters than the former, i.e., approximately 355 M (RoBERTa-large) respectively 333 M (BioLink-BERT-large) vs. 100 M (PubMedBERT). Refer to Appendix 6 for obtaining basic information (e.g. number of parameters and pre-training strategy) of the three PLMs. We selected these models based on previous research (e.g., [4, 43]), in order to support consistency and comparability across studies. We use binary cross entropy as loss function to train the base models. We optimize our model using Adam [27] with a learning rate schedule in which the learning rate is linearly increased from zero to the target learning rate during the first 10% of training steps and then linearly decayed to zero for the remaining 90%. We explore the following hyperparameter options for the base models:

- learning rate: {5e-6, 3e-5, 5e-5}
- maximum sequence length: {256, 384, 512}
- batch-size: {8, 16, 32}
- additional context sentences: {0, 1}
- task description prompt: {yes, no}

Altogether, 108 parameter configurations are evaluated for each PLM and data set. We use the Huggingface transformers [47] and PyTorch Lightning^9^ for implementing our model^10^ and BigBio [18] for accessing the data sets.

#### Model extensions

For assessing the impact of the model extensions, we leverage the best-performing PLM from the previous step and examine the following options depending on the type of additional information:

- *Additional textual information* We test adding only the head entity description, only the tail description, or both to the input text. We append an empty string if the knowledge base does not provide a textual description for a given entity mention. Since the augmentation of the input text increases its length, we also investigate higher maximum lengths, e.g., if the best baseline hyperparameter configuration has a maximum length of 384; we test 384 and 512 as maximal input lengths when adding the entity descriptions.
- *Additional embedded information* We use a two-layer perceptron with hidden and output layer sizes of 100 and 0.2 as dropout probability for integrating the knowledge graph and literature embeddings. We train the MuRE and RotatE knowledge graph embeddings using the PyKeen library [2], optimizing the hyperparameters (e.g., embedding size, learning rate, batch size) on a development set by running and evaluating 100 parameter configurations per model and graph. The link prediction results of the best models on the validation set can be found in Appendix 9. Concerning the literature embeddings, we leverage the pre-computed vectors^11^ and examine all available embedding sizes (i.e., 500, 1000, 1500, and 2000 dimensions). For both embedding types, we explore applying distinct learning rates (i.e., {0.001,0.0001,0.0005}) while training the network.
- *Structural information* We evaluate the following fingerprint methods: atom-pair descriptors, Morgan, and RDK topological fingerprints. Moreover, we build a combined representation by concatenating the three fingerprints. For generating the fingerprints, we leverage the RDKit library.^12^ For encoding the molecular structure by MolBERT and ChemBERTa, we leverage the publicly available pre-trained models of both methods.^13^ We also investigate the impact of applying distinct learning rates (i.e., {0.001,0.0001,0.0005}) for both transformer networks.

In total, we are conducting over 100 experiments to investigate the incorporation of additional context information for each data set.

### Results

Table 3 shows our experimental results. Please refer to Appendix 4.2 for relation type-specific results. We report micro-averaged F1 scores of relation extraction and standard deviation over three runs using different random seeds for each data set and configuration. Detailed results including precision and recall scores per data set can be found in Appendix 8. In the following, we discuss the base model results before reviewing the impact of incorporating the additional context information.

#### Base model results

First, BioLinkBERT-large represents the best-performing model concerning all five data sets, followed by RoBERTa-large and PubMedBERT. However, the differences in performance between BioLinkBERT and RoBERTa are relatively small for three of the five data sets, i.e., BC5CDR, ChemProt, and DDI. For example, for extracting chemical-protein interactions from ChemProt, BioLinkBERT reaches an F1 score of 79.92, whereas the RoBERTa model achieves 79.82. In contrast, the performance differences between the two large models and PubMedBERT are much more pronounced (i.e., PubMedBERT only obtains an F1 score of 77.25 on ChemProt). However, considering the number of parameters (see Appendix 6), these results can certainly be expected, as the two large models have three times as many parameters as the PubMedBERT model (355 M/333 M vs. 100 M). The results achieved for ChemProt and DDI are on par or even slightly better for all three language models compared to those reported in the original publications of the PLMs (see Appendix 6). We attribute the performance differences to a) a broader hyperparameter search during model optimization and b) technical details regarding the representation of the input instances.

In addition to the differences between the models, we also see considerable performance variations depending on the granularity of the annotated relations, i.e., mention- or document-level. For the former, i.e., ChemProt, CPI, and DDI, the models generally reach F1 scores of 80.0 and above, where the scores for the document-level data sets, i.e., BC5CDR and ChemDisGene, do not exceed 68.1. For instance, instead of considering the complete document context when making a decision, we rely on a window of two sentences. However, our results are nonetheless competitive, e.g., for BC5CDR, we reach an F1 score of 68.1, while state-of-the-art approaches designed explicitly for document-level extraction achieve scores of 69.4 [52].

Appendix 7 shows the best-performing hyperparameter setting for each PLM and data set. Note that the best-performing settings show stark variations, both concerning an individual and multiple PLMs. No configuration performs best for at least two data sets, even for a single PLM. Regarding batch size, no PLM achieves the best performance with the lowest batch size evaluated (i.e., 8). Similarly, the best extraction quality is reached with maximum sequence lengths of 256 and 384. Only in one case, RoBERTa-Large with BC5CDR, does a batch size of 512 show the best results. In two-thirds of the cases, including additional context sentences is part of the best configuration, highlighting the positive effect of extending the input text across different PLMs. In contrast, prepending task prompts only benefit the two larger PLMs, BioLinkBERT-large and RoBERTa-large.

**Table 3:**
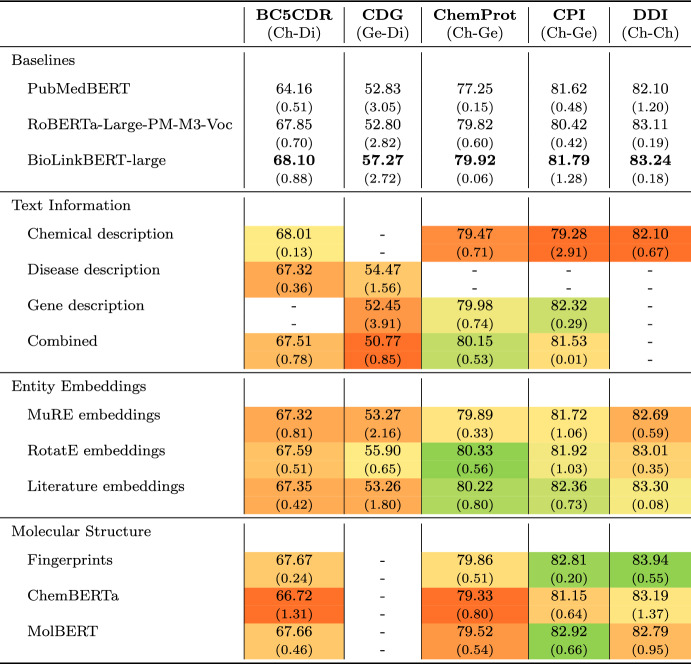
Results of our experimental evaluation

For each data set we highlight the included relation scenario using (Ch) for chemicals, (Di) for diseases, and (Ge) for gene in parenthesis. We perform three runs for each setting and report mean F1 and standard deviation. First, we perform hyperparameter tuning of the three PLM base models and select the best-performing PLM per data set for evaluating the model extensions. For the model extensions, we perform color-coding for each column, i.e., data set, highlighting performance improvements (compared to the best base result) in green and declines in red. The BioLinkBERT-large model performs best across all data sets and relation scenarios

#### Model extensions

The investigated extensions provide only slight result improvements for only a few cases. In most settings, i.e., 30 out of 40 tested configurations, the additional information harms the base model’s performance across all scenarios. Concerning the different types of information, the results show the following impacts:

- *Textual information*: Augmenting the input text with additional entity descriptions does not improve results in 76% of the cases. When using chemical and gene descriptions in ChemProt, the F1 score slightly increases from 79.92 to 80.15. However, in this setup, the variance of the model also increases substantially (SD of 0.53 vs. 0.06), which calls into question the robustness of the improvements achieved. For the other data sets, no benefits can be recorded. For ChemDisGene and DDI, considerable performance declines are recognized, e.g., from 83.24 to 82.10 (−1.4%) when using chemical descriptions in the case of the DDI corpus and from 57.27 to 50.77 (- 7.9%) when using gene and disease definitions in ChemDisGene.
- *Additional entity embeddings*: Augmenting the base model with embedded information provides a similar picture. Utilizing literature embeddings shows minor performance gains for DDI (83.30 vs. 83.24), CPI (81.79 vs. 82.36), and ChemProt (80.22 vs. 79.92). For ChemProt, including knowledge base embeddings learned via RotatE also reaches the best result (80.33 vs. 79.92). Considering the models’ variance, however, these gains are likely attributable to statistical fluctuations. For BC5CDR and ChemDisGene, no performance gains can be recognized. Especially for the latter, sharp performance drops are recognized, e.g., augmenting the model with literature embeddings reaches an F1 score of 53.36, representing a decline by 4 pp (−7%) compared to the base model.
- *Structural information*: The extension of the model by integrating molecular structure information positively impacts relation identification in CPI and DDI. Concerning DDI, the model using molecular fingerprints performs best, reaching an F1 score of 83.94, representing an improvement of 0.7 pp compared to the base model. Interestingly, the more advanced transformer-based language models, MolBERT and ChemBERTa, do not yield such benefits. For CPI, using fingerprints and MolBERT-based embeddings shows considerable performance benefits (82.81 and 82.92 vs. 81.79). Again, no improvements can be recorded for the other relation extraction use cases.

## Discussion

### Impact of the language model

Our experimental results mainly confirm the findings in [43], showing only minor performance enhancements using additional context information. However, they contradict several other studies that report considerable performance improvements when integrating entity information into PLM-based models [5, 6, 39]. One important difference between these studies and ours concerns the language models used as a base model. Studies reporting performance improvements leverage either PubMedBERT [20], BioBERT [30] or SciBERT [8], In contrast, we also include larger PLMs in our evaluation. To gain insights into the influence of the choice of language model on the results achieved when augmenting the PLMs, we carried out additional experiments using only PubMedBERT (recall that the results reported in Table 3 were obtained with using optimized models per setting, mostly relying on BioLinkBert-Large as base model).

Table 4 contains the results achieved in this setting, contrasting those obtained using the BioLinkBERT-large model. Clear performance benefits can be recognized across all five data sets. The augmentation of the model with entity embeddings highlights the most substantial gains. Integrating the literature embeddings into the fine-tuning process works best, showing an average improvement of 0.9 pp in F1. When excluding ChemDisGene, for which no improvements can be achieved, the mean score increases by an average of 1.18 pp. Incorporating MuRE and RotatE knowledge graph embeddings represents the second and third-best configurations.

Using molecular structure information improves the extraction quality for BC5CDR and ChemProt. For instance, leveraging the ChemBERTa model reaches an F1 score of 65.66 (+ 1.5 pp) and 78.08 (+ 0.83 pp) for BC5CDR and ChemProt, respectively. Interestingly, similar to the results using BioLinkBERT, we see no improvements for CPI, although the data set also contains chemical-protein interactions like ChemProt. Concerning the textual context information, performance benefits can be recorded only for ChemProt. In this case, adding gene descriptions to the input text reaches a score of 78.22, representing an increase of 0.97 pp compared to the base model. Integrating chemical descriptions achieves slightly better results (77.7, + 0.45 pp). For the other data sets, however, textual descriptions harm the extraction performance of PubMedBERT.

To summarise, when using PubMedBERT als PLM, our study essentially supports the findings from previous studies [5, 6, 39]. These results indicate that the larger PLMs implicitly encode (to some extent) the supervision signals obtained by the additional context information, and that the apparent contradictions in observations between our study and prior work probably can be explained by the different numbers of model parameters in the base language models.

**Table 4:**
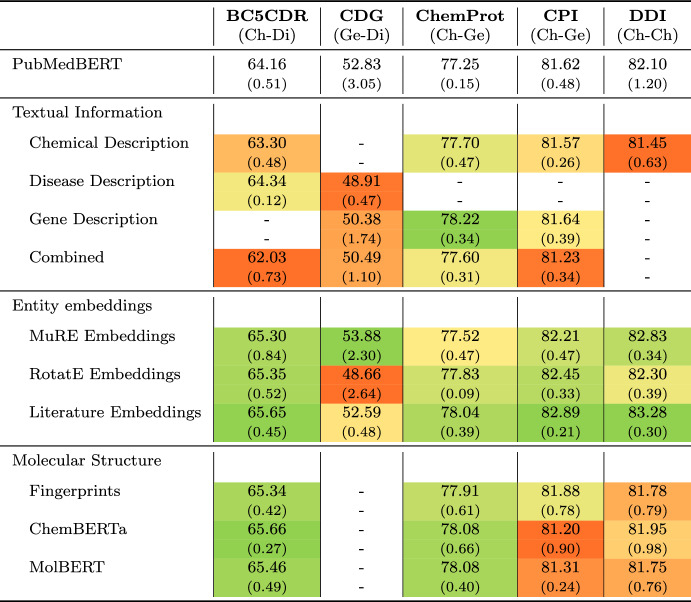
Results of our experimental evaluation using PubMedBERT as underlying baseline model

For each data set we highlight the included relation scenario using (Ch) for chemicals, (Di) for diseases, and (Ge) for genes. We perform three runs for each setting and report mean F1 and standard deviation (in parenthesis below). For the model extensions, we perform color-coding for each column, i.e., data set, highlighting performance improvements (compared to the baseline result) in green and declines in red

### Fine-grained relation type results

The gold-standard annotations of the ChemProt and DDI corpus distinguish between five types of chemical-protein interactions and four types of drug-drug interactions, respectively. The results in Sect.  3.2 only show an aggregated picture of the performance of the different PLMs and the extensions investigated, not accounting for relation type-specific performance differences in these two scenarios. We also investigated the type-specific scores to gain a more fine-grained understanding of how well the PLMs and the proposed model extensions perform. For this experiment, we used the best-performing PLM for each scenario, i.e., BioLinkBERT-large, and the best-performing configuration for each type of model extension, i.e., textual side information, embedded side information, and structure information. We excluded the ChemDisGene data set, which distinguishes two types of gene-disease interactions due to its small size.

Table 5 highlights the relation type-specific results that vary considerably between individual relation types, e.g., from 70.88 (*Substrate*) to 84.51 (*Downregulator*) for ChemProt and from 58.37 (*Interaction*) to 90.44 (*Advise*) for DDI in case of the baseline model. Regarding the ChemProt results, it should first be noted that BioLinkBERT-large achieves surprisingly good results even for relation types with low support. For *Antagonist*, which represents the type with the second-fewest instances, the model achieves the second-best score (83.68). However, the highest score is reached for the majority type *Downregulator*. When we inspect the results of the model extensions, we see a rather heterogeneous picture concerning the different types of enhancements. Text and structure information are beneficial for the low-support relation types. For instance, augmenting the model with structure information improves the F1 score by 0.56 pp for *Agonist* and 1.57 pp for *Antagonist*. In contrast, utilizing embedded context information is advantageous for *Substrate* relations, lifting the score by 1.31 pp. These diverse results suggest that ensembling models with different extension types could lead to improvements across different relation types.

The picture of the results for DDI is different. Performance increases can primarily be reached for *Effect* relations by enhancing the model with embedded entity (81.57, + 0.75 pp) and structure information (81.55, + 0.73 pp). Using additional textual descriptions provides no performance boosts for any type of interaction. Moreover, the extraction of *Mechanism* and *Advice* is not improved by any additional context information. Sometimes, even sharp performance declines are recognized, e.g., $-$1.5 pp for *Mechanism* interactions when using textual entity descriptions.

**Table 5:**
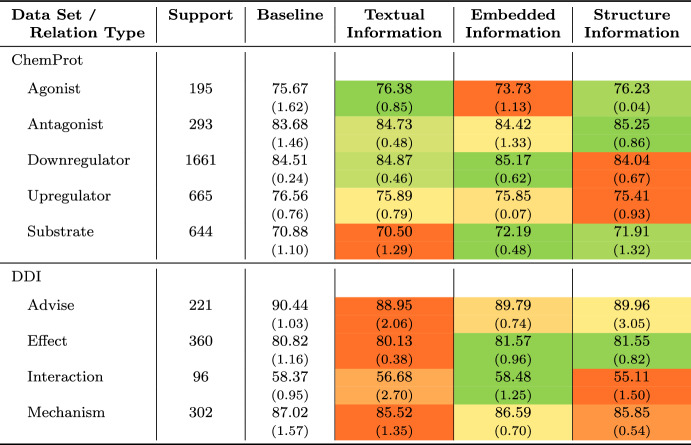
Relation type-specific evaluation results of the best baseline model, i.e., BioLinkBERT-large, and the best-performing model enhancement per type of context information (i.e. textual, embedded and structure information)

For each setting we report mean F1 and standard deviation (in parenthesis below) over the three runs performed. For each row, we apply color-coding indicating performance increases (green) and declines (green) compared to the baseline model results

### Impact of training size

When fine-tuning PLMs for relation extraction, a constraint is the requirement for (high-quality) training data [34]. The annotation of relational information in biomedical texts is a complex task requiring extensive domain expertise and is, therefore, time-consuming and costly to produce [24]. The data sets we analyzed have between 523 (ChemDisGene) and 2432 (ChemProt) instances for training and evaluating the models (see Appendix 1). These considerations raise the question of how the models’ performance relates to the underlying amount of training data available. Moreover, the amount of available data can influence how the effectiveness of additional contextual information is interpreted when comparing results across studies using different datasets. To better understand this relationship, we tested the performance of the three baseline PLM models having access only to a limited number of documents from the training set, i.e., 25, 50, 75, ..., 200 training instances. We perform six runs for each training set size and PLM using different random seeds and report mean performance of all runs. We set the hyperparameters of the models to the best found during our original evaluation. When building the partial training splits, we ensure a similar label distribution compared to the complete training set for data sets distinguishing multiple relation types.

**Fig. 2:**
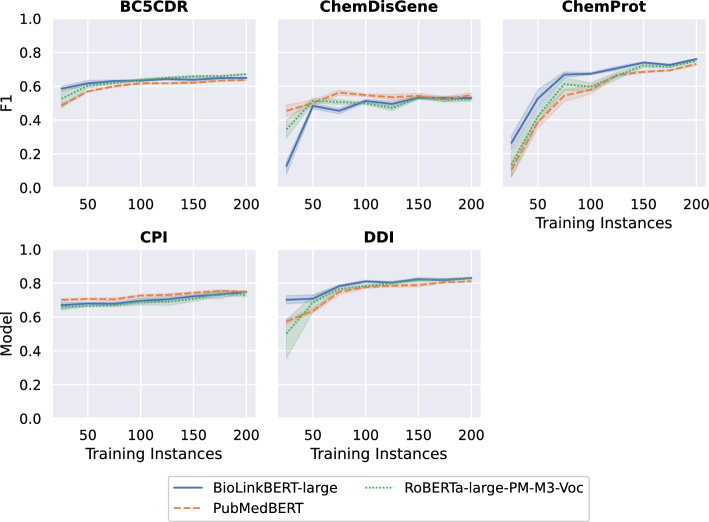
Evaluation results of the models in scenarios with a reduced amount of training data. We perform six runs for each training set size and language model using different random seeds and report mean performance of all runs. For three of the five data sets tested, i.e., BC5CDR, CPI, and DDI, the models achieved promising results even with just 25 training documents. With ChemProt and ChemDisGene, the models benefit substantially from having access to more training data

Figure 2 illustrates the obtained results showing notable differences between the relation scenarios and data sets. Steep increases in performance with increasing amounts of training data are particularly evident for extracting chemical-protein interactions in ChemProt and disease-gene relations in ChemDisGene. For example, BioLinkBERT reaches an average F1 score of 27.15 when using only 25 documents from ChemProt for training, representing 34% of the accuracy compared to the default evaluation on the entire data set. Similarly, for ChemDisGene, the model achieves a score of 12.82 (22.4%) when trained on 25 documents. However, performance improves sharply in this scenario when training on 50 documents, achieving an average score of 48.35, i.e., almost four times as high. The performance curves of the models for the other data sets are smoother. In the case of CPI, for instance, the models already achieve accuracies between 82% (RoBERTa-large-PM-M3-Voc) and 85.8% (PubMedBERT) of their full-corpus performance when trained on only 25 documents.

Concerning the ranking of the three PLM models, it is noteworthy that the results obtained during the regular evaluation (see Table 3) are only consistent for three of the five data sets in the restricted evaluation setting. For ChemDisGene and CPI, the PubMedBERT model, which has the fewest parameters of the investigated PLMs, achieves better results than the two larger models when trained on less than 150 instances. For example, in the case of CPI, PubMedBERT outperforms BioLinkBERT and RoBERTa-large-PM-M3-Voc by 2.7 pp and 4.3 pp, respectively, reaching an F1 score of 70.7 when restricting the training set to 50 documents.

To summarise, the sensitivity of the achieved performance to the amount of the underlying training data depends strongly on the relation scenario and the data set used. For three of the five data sets tested, i.e., BC5CDR, CPI, and DDI, the models achieved promising results even with just 25 training documents. With ChemProt and ChemDisGene, the models benefit substantially from having access to more training data. These results indicate that differences in performance reported in prior works cannot be explained (or only to a small extent) by the size of the training data set used. Finally, none of the performance curves in Figure 2 converges, highlighting the potential for further performance improvements by additional training instances.

### Sentence versus document-level relation extraction

Two of the used data sets, i.e., BC5CDR and ChemDisGene, contain relation annotations given the context of the entire document (*document-level*) rather than identifying relations between individual pairs of entity mentions (*mention-level*). These differences in annotation granularity usually lead to different choices when designing a model [50]. Accordingly, methods for relation extraction are categorized into sentence-level and document-level approaches [14, 50]. Our approach is focused on sentence-based extraction, which limits its effectiveness for document-level data sets such as BC5CDR and ChemDisGene. Both data sets provide entity annotations on the mention level, comprising MESH identifiers for each entity mention, but have relation annotations only at the document level. There is no annotated information telling which sentence and which specific pair of mentions express the relationship between two entities. To compensate for this situation, we build an input instance for each pair of entities mentioned within a window of two sentences when evaluating BC5CDR and ChemDisGene. Moreover, we compute the union of all predicted relation types for mention pairs referring to the same entities (according to their MESH identifiers) as the document-level prediction. To quantify the number of missed relationships of our approach, we analyze the minimal sentence distance for each relation contained in the corpora. For each gold standard pair, we collect all mentions of the head and tail entity and compute the shortest distance between every instance of the Cartesian product of both mention sets. We found that 96.34% of the ChemDisGene and 94.16% of the BC5CDR relations are within a context of two sentences, illustrating an upper bound of our approaches’ performance.

Considering the results achieved (see Table 3), the models show large performance differences between the document- and sentence-level data sets. For instance, the best BioLink-BERT-large base model reaches a mean F1 score of 62.69 for the two document-level and 81.65 for the three sentence-level data sets. To gain deeper insights into the predictions for the document-level data sets, we examined how the model’s recall varies with the minimum sentence distance between the mentions of the two entities forming a gold-standard pair. For BC5CDR, the model achieves a recall of 0.84 for intra-sentence pairs. In contrast, this value decreases to 0.52 for inter-sentence relations, which consist of entity pairs spanning two or more sentences. Similarly, in the case of ChemDisGene, the model reaches 0.67 for intra- and 0.42 for inter-sentence relations. These findings suggest that the models encounter difficulties when dealing with more extended textual context and potential inter-sentence dependencies, highlighting the challenge of effectively capturing long-range relations.

### Prediction analysis

Our evaluation demonstrates that incorporating additional knowledge yields positive effects only in selected scenarios (see Table 3). This raises the question of the specific circumstances that are contributing to these improvements. A hypothesis is that the inclusion of additional information is particularly beneficial for instances involving entities that are not present in the training split as the model does not receive any supervision signal for these cases. We analyzed the model predictions for configurations that demonstrated improved extraction performance. In our evaluation, each configuration was run three times using different random seeds. To ensure a fair comparison and obtain a more representative assessment, we report results based on the second-best performing model for both the baseline (i.e., BioLinkBERT-large) and each respective model extension, thereby excluding the best and worst model per configuration. For entity identification, we leverage the corresponding knowledge base identifier for each entity.

Table 6 presents, for each dataset and configuration, the number of instances misclassified by the baseline model but correctly predicted by the extended model. Moreover, the table highlights the proportion of these cases constituting zero-shot examples, i.e., instances in which the head entity (*new head*), the tail entity (*new tail*), or both are not present in the training set. The results show that 34.5â€“69.4% of the observed improvements can be attributed to zero-shot instances. The phenomenon is more pronounced for the ChemProt and DDI corpora. For the former, using both textual chemical and gene descriptions results in 25 out of 36 corrected instances being zero-shot examples. For the latter, when molecular fingerprints are used, 60% of the observed improvements can be attributed to these instances. In case of CPI, the ratios of zero-shot instances are lower. The highest value is observed when molecular fingerprints are utilized, with 27 out of 58 corrected instances representing zero-shot examples. These results imply that additional information may enhance the model’s performance on unseen entities. However, due to the limited number of examples, these findings should be interpreted with caution and warrant further investigation on a larger data base.

**Table 6:** Overview about the number of instances misclassified by the baseline model but correctly predicted by the extended model (referred as *improved instances*) for configurations with improved performance

Data set/model extension	Improved predictions	Zero-shot examples			
		Total	Unseen head	Unseen tail	Unseen both
ChemProt					
Rotate embeddings	28	17	13	13	9
Literature embeddings	32	19	14	15	10
Chemical & gene descriptions	36	25	17	18	10
Gene descriptions	31	18	13	13	8
CPI					
MolBERT	58	20	17	8	5
Molecular fingerprints	55	27	19	12	4
Gene descriptions	59	27	21	10	4
Literature embeddings	45	19	16	9	6
DDI					
Molecular fingerprints	15	9	4	5	0

Moreover, we report the number of improved instances that constitute zero-shot examples, i.e., cases where the head entity (*unseen head*), the tail entity (*unseen tail*), or both are not present in the training set. For entity identification, we leverage the corresponding knowledge base identifier for each entity. The configurations are ordered based on their test set performance (see Table 3)

## Conclusion

In our study, we evaluated transformer-based language models augmented with additional context information across various biomedical relation scenarios, using five distinct datasets within a unified framework, a benchmark desperately missing so far. We conducted thorough hyper-parameter tuning before enhancing the best-performing models with additional entity and molecular information. Our results show that the BioLinkBERT-large model achieved state-of-the-art performance in multiple scenarios, though the overall benefits of including extra context were minimal. An ablation study further demonstrated that models with fewer parameters significantly benefit from this additional information during fine-tuning.

## Data Availability

The datasets employed are publicly available as detailed in the following publications: $\bullet$ BioCreative-V-CDR (BC5CDR) [32] $\bullet$ ChemProt [28], $\bullet$ CPI [15] $\bullet$ ChemDisGene [49] $\bullet$ DDI corpus [23]

## Appendix 1 data sets

**BC5CDR**: For chemical-disease relationships, we leverage the BioCreative V CDR corpus (BC5CDR) [32] containing 1500 PubMed articles with 15,953 annotated chemicals, 13,318 diseases, and 3169 chemical-disease interactions. The data set provides entity identifiers for both entity types using Medical Subject Headings (MeSH) on mention level. In contrast, relation annotations are given only on document-level, i.e., instead of identifying relations between individual pairs of entity mentions (*mention-level*), the corpus provides chemical-disease interactions within the context of entire documents. Moreover, the data set does not discern different subtypes of chemical-disease relations.

**ChemProt**: We use the ChemProt corpus [28], created in the context of the BioCreative Challenge VI in 2016. The data set consists of 2482 scientific abstracts from PubMed and identifies 32,514 chemical and 30,922 protein mentions and their interactions, differentiated into nine different types. Within the evaluation of the challenge, these interaction types were divided into five groups (see Table 8). In our approach, we follow the evaluation protocol of the challenge by using the five interaction groups as gold standard annotations.

**CPI**: As a second data set for chemical-protein interactions, we utilize the relations given in the compound-protein corpus (CPI) [15]. The data set consists of 2613 sentences from 1808 PubMed abstracts containing 3563 chemical and 4256 protein mentions. In total, 2931 chemical-protein interactions are annotated. In contrast to the ChemProt data set, no distinct types of chemical-protein interactions are distinguished.

**ChemDisGene**: For evaluating the extraction of gene-disease relationships, we use the curated corpus provided by the ChemDisGene data set [49] containing 523 scientific abstracts from PubMed. The data set comprises 5739 chemicals, 2931 diseases, and 5578 gene annotations and interactions between them. Chemicals and diseases are normalized to CTD chemicals and CTD diseases using MESH identifiers. Genes are identified using NCBI Gene. Our study uses the 576 document-level gene-disease associations from the data set, further categorized into *marker/mechansim* and *therapeutic* relations.

**DDI corpus**: For the identification of drug-drug interactions, we utilize the DDI corpus [23], which contains 158 MedLine abstracts and 683 texts from the DrugBank database describing over 5000 interactions between 17,783 drug mentions. The data set distinguishes four types of drug-drug interactions: *mechanism*, *effect*, *advice*, and *interaction*, whereby the last type represents a general relationship between two drugs without a more detailed description.

## Appendix 2 entity normalization statistics

See Table 7.

**Table 7:** Overview of the annotations of BC5CDR, CPI and DDI that we could map to shared ontologies, i.e., NCBI Gene [11] for genes and CTD Chemicals [13] for chemicals

Data set	Entity type	Entitiy mentions		Unique entities	
		Normalized	Ratio (%)	Normalized	Ratio (%)
ChemProt	Chemical	25.566	78.63	5.297	69.35
	Gene/Protein	22.268	72.01	5.415	56.84
CPI	Chemical	3.306	92.79	1.326	91.45
	Gene/Protein	3.092	72.65	1.162	70.90
DDI	Chemical	15.698	88.28	2.528	73.57

For each data set and entity type we report the number of normalized mentions and unique entities (identified by distinct strings) as well as their ratio with regard to the total number of entities in the data set

## Appendix 3 relation labels

See Table 8.

**Table 8:** Overview about the relation and interaction types of the ChemProt corpus [28]

Relation	Relation label	Interaction types
CPR:3	Upregulator	upregulator, activator, indirect upregulator
CPR:4	Downregulator	downregulator, inhibitor, indirect downregulator
CPR:5	Agonist	agonist, agonist-activator, agonist-inhibitor
CPR:6	Antagonist	antagonist
CPR:9	Substrate	substrate, product of

## Appendix 4 textual information

See Table 9.

**Table 9:** Number of entity mentions per data set for which additional textual descriptions could be found in the respective knowledge base

Data Set	Chemicals	Diseases	Genes
BC5CDR	15,210	13,192	
	(95.34%)	(99.05%)	
ChemDisGene		2,836	5,437
		(96.76%)	(97.48%)
ChemProt	24,892		20,574
	(78,20%)		(67,87%)
CPI	3.306		3.092
	(92,79%)		(72.65%)
DDI corpus	16,301		
	(88.16%)		

Note that the knowledge bases do not necessarily contain a textual description for each entity they list. Hence, we can not extract textual descriptions for each entity even if the data set provides entity identifiers (e.g., BC5CDR and ChemDisGene)

Numbers in parentheses indicate the size of the splits we created when the dataset does not provide them

## Appendix 5 knowledge graph statistics

See Table 10

**Table 10:** Statistics of the CTD-based knowledge graphs that we use for training knowlege base embeddings using MuRE [7] and RotatE [40]

	Knowledge graph		
	Complete	Chemical-disease	Chemical-gene
Triples	1,131,375	1,033,060	78,272
Entities			
Chemicals	16,265	15,752	7,646
Diseases	7,068	7,071	–
Genes	15,097	–	15,176
Phenotypes	2,797	–	–
Total	41,227	22,823	22,822
Relation types	124	1	115

Note, the high number of relation types for the complete and the chemical-gene graph results from the fine-granular modelling of relationships in CTD, e.g., for many interactions also the effect is recorded in the relation type (e.g., *increases-expression* and *decreases-expression*)

## Appendix 6 transformer models

See Table 11

**Table 11:** Overview of the evaluated language models. For each model, we report the number of parameters and pre-training information

	PubMedBERT	RoBERTa-Large- PM-M3-Voc	BioLinkBERT -large
	[20]	[31]	[48]
Parameters	$\sim$ 100M	$\sim$ 355M	$\sim$ 333M
Pre-training			
Data	PubMed	PubMed+PMC+MIMIC-III	PubMed
Method	from scratch	from scratch	from scratch
Tasks	MLM+NSP	MLM+NSP	MLM+NSP+DRP
RE results			
ChemProt	77.24	76.2	79.98
DDI	82.36	82.1	83.35

Moreover, we include the relation extraction results reported in the original article of each PLM for data sets used in this study. Pre-training tasks include masked language modeling (MLM), next sentence prediction (NSP), and document relation prediction (DRP). All models are pre-trained from scratch instead of continuing training of existing PLMs

## Appendix 7 best-performing hyperparameters

See Table 12

**Table 12:** Best-performing hyperparameter setting per PLM and data set

PLM/data set	Learning rate	Batch size	Max. sequence length	Context size	Task prompt
PubMedBERT					
BC5CDR	0.00003	32	256	1	–
ChemDisGene	0.00005	32	384	0	–
ChemProt	0.00005	32	384	1	–
CPI	0.00005	16	256	0	–
DDI	0.00005	32	256	1	–
RoBERTa-Large-PM-M3-Voc					
BC5CDR	0.000005	32	512	1	$\checkmark$
ChemDisGene	0.00003	32	384	1	–
ChemProt	0.00003	32	256	1	–
CPI	0.00003	16	256	0	$\checkmark$
DDI	0.00003	32	384	1	–
BioLinkBERT-large					
BC5CDR	0.00003	16	384	1	$\checkmark$
ChemDisGene	0.000005	16	384	1	–
ChemProt	0.00005	32	256	1	$\checkmark$
CPI	0.00005	16	256	0	$\checkmark$
DDI	0.00003	32	384	0	–

## Appendix 8 Fine-grained results per data set

### Appendix 8.1 BC5CDR

See Table 13

**Table 13:** Detailed results of our experimental evaluation on BC5CDR

	BC5CDR			
	P	R	F1	$\sigma$
Baselines				
PubMedBERT	57.99	71.94	64.16	0.51
RoBERTa-Large-PM-M3-Voc	63.35	73.10	67.85	0.70
**BioLinkBERT-large**	**61.73**	**75.98**	**68.10**	**0.88**
Text				
Chemical Description	62.53	74.69	68.01	0.13
Disease Description	64.37	70.74	67.32	0.36
Combined	63.09	72.61	67.51	0.78
Entity				
MuRE Embeddings	64.07	70.99	67.32	0.81
RotatE Embeddings	62.75	73.25	67.59	0.51
Literature Embeddings	61.74	74.14	67.35	0.42
Structure				
Fingerprints	62.27	74.11	67.67	0.24
ChemBERTa	61.08	73.53	66.72	1.31
MolBERT	61.80	74.82	67.66	0.46

We perform three runs for each setting and report mean precision, recall and F1 as well as F1 standard deviation. The best-performing baseline is highlighted bold

### Appendix 8.2 ChemDisGene

See Table 14

**Table 14:** Detailed results of our experimental evaluation on ChemDisGene

	ChemDisGene			
	P	R	F1	$\sigma$
Baselines				
PubMedBERT	44.54	65.97	52.83	3.05
RoBERTa-Large-PM-M3-Voc	45.78	62.94	52.80	2.82
**BioLinkBERT-large**	**51.77**	**64.34**	**57.27**	**2.72**
Text				
Disease Description	49.82	60.14	54.47	1.56
Gene Description	47.05	59.44	52.45	3.91
Combined	45.67	57.58	50.77	0.85
Entity				
MuRE Embeddings	49.90	58.28	53.27	2.16
RotatE Embeddings	50.33	62.94	55.90	0.65
Literature Embeddings	50.23	57.58	53.26	1.80

We perform three runs for each setting and report mean precision, recall and F1 as well as F1 standard deviation. The best-performing baseline is highlighted bold

### Appendix 8.3 ChemProt

See Table 15

**Table 15:** Detailed results of our experimental evaluation on ChemProt

	Chemprot			
	P	R	F1	$\sigma$
Baselines				
PubMedBERT	77.98	76.55	77.25	0.15
RoBERTa-Large-PM-M3-Voc	80.99	78.74	79.82	0.60
**BioLinkBERT-large**	**79.29**	**80.57**	**79.92**	**0.06**
Text				
Chemical Description	79.75	79.18	79.47	0.71
Gene Description	80.28	79.81	79.98	0.74
Combined	80.95	79.38	80.15	0.53
Entity				
MuRE Embeddings	80.31	79.47	79.89	0.33
RotatE Embeddings	81.50	79.20	80.33	0.56
Literature Embeddings	80.11	80.33	80.22	0.80
Structure				
Fingerprints	78.11	81.68	79.86	0.51
ChemBERTa	80.31	78.41	79.33	0.80
MolBERT	80.15	78.93	79.52	0.54

We perform three runs for each setting and report mean precision, recall and F1 as well as F1 standard deviation. The best-performing baseline is highlighted bold

### Appendix 8.4 CPI

See Table 16

**Table 16:** Detailed results of our experimental evaluation on CPI

	CPI			
	P	R	F1	$\sigma$
Baselines				
PubMedBERT	78.13	85.46	81.62	0.48
RoBERTa-Large-PM-M3-Voc	75.11	86.58	80.42	0.42
**BioLinkBERT-large**	**76.83**	**87.55**	**81.79**	**1.28**
Text				
Chemical Description	72.94	86.88	79.28	2.91
Gene Description	77.87	87.32	82.32	0.29
Combined	76.44	87.40	81.53	0.01
Entity				
MuRE Embeddings	75.67	88.81	81.72	1.06
RotatE Embeddings	77.03	87.47	81.92	1.03
Literature Embeddings	76.42	89.41	82.36	0.73
Structure				
Fingerprints	78.04	88.22	82.81	0.20
ChemBERTa	75.26	88.07	81.15	0.64
MolBERT	78.91	87.40	82.92	0.66

We perform three runs for each setting and report mean precision, recall and F1 as well as F1 standard deviation. The best-performing baseline is highlighted bold

### Appendix 8.5 DDI

See Table 17

**Table 17:** Detailed results of our experimental evaluation on DDI.

	DDI			
	P	R	F1	$\sigma$
Baselines				
PubMedBERT	81.29	82.94	82.10	1.20
RoBERTa-Large-PM-M3-Voc	83.11	83.11	83.11	0.19
**BioLinkBERT-large**	**83.49**	**83.01**	**83.24**	**0.18**
Text				
Chemical Description	83.06	81.24	82.10	0.67
Entity				
MuRE Embeddings	82.28	83.11	82.69	0.59
RotatE Embeddings	82.85	83.18	83.01	0.35
Literature Embeddings	83.49	83.11	83.30	0.08
Structure				
Fingerprints	83.92	83.96	83.94	0.55
ChemBERTa	82.11	84.34	83.19	1.37
MolBERT	83.35	82.26	82.79	0.95

We perform three runs for each setting and report mean precision, recall and F1 as well as F1 standard deviation. The best-performing baseline is highlighted bold

## Appendix 9 knowledge graph embedding results

See Table 18

**Table 18:** Validation set link prediction performance for RotatE [40] and MuRE [7] on the different CTD-based knowledge graphs used (see Sect.  2.3)

Method / Knowledge Graph	Head		Tail		Both	
	Hits@10	MR	Hits@10	MR	Hits@10	MR
RotatE [40]						
CTD-Chemical-Disease	0.066	854	0.130	296	0.098	497
CTD-Chemical-Gene	0.246	1104	0.239	1769	0.243	1304
CTD Complete	0.053	1041	0.098	369	0.075	608
MuRE [7]						
CTD-Chemical-Disease	0.049	793	0.120	245	0.085	445
CTD-Chemical-Gene	0.163	441	0.151	1594	0.157	751
CTD Complete	0.034	1066	0.089	328	0.062	595

For each model and knowledge graph, results are reported using Hits@10 and median rank (MR) metrics, across head entity prediction, tail entity prediction, and combined evaluations. All performance metrics reflect a realistic evaluation protocol (refer to [3])

## Footnotes

1 CTD Diseases is also known as MEDIC and represents a subset of MeSH [33] and OMIM [21].

2 CTD chemicals represents a subset of MESH [33].

3 https://www.uniprot.org/id-mapping.

4 https://ftp.ncbi.nih.gov/pubchem/Compound/Extras/CID-MeSH.

5 http://ctdbase.org/reports/CTD_chem_gene_ixns.tsv.gz.

6 https://ctdbase.org/reports/CTD_chemicals_diseases.tsv.gz.

7 https://ctdbase.org/reports/CTD_pheno_term_ixns.tsv.gz.

8 MolBERT: https://github.com/BenevolentAI/MolBERT; ChemBERTa: https://github.com/seyonechithrananda/bert-loves-chemistry.

9 https://github.com/Lightning-AI/pytorch-lightning.

10 Model code available at https://github.com/mariosaenger/biore-kplm-benchmark.

11 Available at https://github.com/mariosaenger/bio-re-with-entity-embeddings.

12 https://www.rdkit.org/.

13 ChemBERTa: https://huggingface.co/seyonec/PubChem10M_SMILES_BPE_450k and MolBERT: https://ndownloader.figshare.com/files/25611290.

## Code availability

The experiment code is available at https://github.com/mariosaenger/biore-kplm-benchmark.

## References

[1] Aldahdooh J, Tanoli Z, Tang J. Mining drug-target interactions from biomedical literature using chemical and gene descriptions-based ensemble transformer model. Bioinform Adv. 2024;4(1):106. 10.1093/bioadv/vbae106 PMC11293871 39092007

[2] Ali M, Berrendorf M, Hoyt CT, et al. Pykeen 1.0: a python library for training and evaluating knowledge graph embeddings. J Mach Learn Res. 2021;22(82):1–6.

[3] Ali M, Berrendorf M, Hoyt CT, et al. Bringing light into the dark: a large-scale evaluation of knowledge graph embedding models under a unified framework. IEEE Trans Pattern Anal Mach Intell. 2022;44(12):8825–45. 34735335 10.1109/TPAMI.2021.3124805

[4] Asada M, Gunasekaran N, Miwa M, et al. Representing a heterogeneous pharmaceutical knowledge-graph with textual information. Front Res Metrics Anal. 2021;6:670206. 10.3389/frma.2021.670206 PMC8281808 34278204

[5] Asada M, Miwa M, Sasaki Y. Using drug descriptions and molecular structures for drug-drug interaction extraction from literature. Bioinformatics. 2021;37(12):1739–46. 33098410 10.1093/bioinformatics/btaa907 PMC8289381

[6] Asada M, Miwa M, Sasaki Y. Integrating heterogeneous knowledge graphs into drug-drug interaction extraction from the literature. Bioinformatics. 2023;39(1):754. 10.1093/bioinformatics/btac754 PMC9805562 36416141

[7] Balazevic I, Allen C, Hospedales T. Multi-relational poincaré graph embeddings. Adv Neural Inf Process Syst. 2019;32.

[8] Beltagy I, Lo K, Cohan A. Scibert: A pretrained language model for scientific text. In: Inui K, Jiang J, Ng V, et al (eds) proceedings of the 2019 conference on empirical methods in natural language processing and the 9th international joint conference on natural language processing, EMNLP-IJCNLP 2019. Association for Computational Linguistics; 2019. pp. 3613–3618.

[9] Bender A, Mussa HY, Glen RC, et al. Molecular similarity searching using atom environments, information-based feature selection, and a naive bayesian classifier. J Chem Inf Comput Sci. 2004;44(1):170–8. 14741025 10.1021/ci034207y

[10] Bolton EE, Wang Y, Thiessen PA, et al (2008) Pubchem: integrated platform of small molecules and biological activities. In: Annual reports in computational chemistry. Elsevier; 2008. vol. 4 , pp. 217–241.

[11] Brown GR, Hem V, Katz KS, et al. Gene: a gene-centered information resource at ncbi. Nucleic Acids Res. 2015;43(D1):D36–42. 25355515 10.1093/nar/gku1055 PMC4383897

[12] Chithrananda S, Grand G, Ramsundar B. Chemberta: large-scale self-supervised pretraining for molecular property prediction. 2020. arXiv preprint arXiv:2010.09885.

[13] Davis AP, Wiegers TC, Johnson RJ, et al. Comparative toxicogenomics database (ctd): update 2023. Nucleic Acids Res. 2023;51:D1257–62. 10.1093/nar/gkac833. 36169237 10.1093/nar/gkac833 PMC9825590

[14] Delaunay J, Tran THH, González-Gallardo CE, et al. A comprehensive survey of document-level relation extraction. 2023. 10.48550/arXiv.2309.16396arXiv preprint arXiv:abs/2309.16396.

[15] Döring K, Qaseem A, Becer M, et al. Automated recognition of functional compound-protein relationships in literature. PLoS ONE. 2020;15(3):e0220925. 32126064 10.1371/journal.pone.0220925 PMC7053725

[16] Dou M, Ding J, Chen G, et al. Ik-ddi: a novel framework based on instance position embedding and key external text for ddi extraction. Brief Bioinform. 2023;24(3):bbad099. 36932655 10.1093/bib/bbad099

[17] Fabian B, Edlich T, Gaspar H, et al. Molecular representation learning with language models and domain-relevant auxiliary tasks. 2020. p. 13230. arXiv preprint arXiv:2011.13230.

[18] Fries J, Weber L, Seelam N, et al. Bigbio: a framework for data-centric biomedical natural language processing. Adv Neural Inf Process Syst. 2022;35:25792–806.

[19] Gaulton A, Bellis LJ, Bento AP, et al. Chembl: a large-scale bioactivity database for drug discovery. Nucleic Acids Res. 2012;40(D1):D1100–7. 21948594 10.1093/nar/gkr777 PMC3245175

[20] Gu Y, Tinn R, Cheng H, et al. Domain-specific language model pretraining for biomedical natural language processing. ACM Trans Comput Healthcare. 2021;3(1):1–23.

[21] Hamosh A, Scott AF, Amberger JS, et al. Online mendelian inheritance in man (omim), a knowledgebase of human genes and genetic disorders. Nucleic Acids Res. 2005;33:D514–7. 15608251 10.1093/nar/gki033 PMC539987

[22] Harpaz R, Callahan A, Tamang S, et al. Text mining for adverse drug events: the promise, challenges, and state of the art. Drug Saf. 2014;37:777–90. 25151493 10.1007/s40264-014-0218-z PMC4217510

[23] Herrero-Zazo M, Segura-Bedmar I, Martínez P, et al. The ddi corpus: an annotated corpus with pharmacological substances and drug-drug interactions. J Biomed Inform. 2013;46(5):914–20. 23906817 10.1016/j.jbi.2013.07.011

[24] Huang Y, Gottardo R. Comparability and reproducibility of biomedical data. Brief Bioinform. 2013;14(4):391–401. 23193203 10.1093/bib/bbs078 PMC3713713

[25] Irwin JJ, Sterling T, Mysinger MM, et al. Zinc: a free tool to discover chemistry for biology. J Chem Inf Model. 2012;52(7):1757–68. 22587354 10.1021/ci3001277 PMC3402020

[26] Kim S, Chen J, Cheng T, et al. Pubchem 2019 update: improved access to chemical data. Nucleic Acids Res. 2019;47(D1):D1102–9. 30371825 10.1093/nar/gky1033 PMC6324075

[27] Kingma DP, Ba J. Adam: a method for stochastic optimization. In: Bengio Y, LeCun Y, (eds). 3rd international conference on learning representations (ICLR); 2015.

[28] Krallinger M, Rabal O, Akhondi SA, et al. Overview of the biocreative vi chemical-protein interaction track. In: Proceedings of the sixth biocreative challenge evaluation workshop; 2017. pp. 141–146.

[29] Lai PT, Wei CH, Luo L, et al. Biorex: improving biomedical relation extraction by leveraging heterogeneous datasets. J Biomed Inform. 2023;146:104487. 37673376 10.1016/j.jbi.2023.104487

[30] Lee J, Yoon W, Kim S, et al. Biobert: a pre-trained biomedical language representation model for biomedical text mining. Bioinformatics. 2020;36(4):1234–40. 31501885 10.1093/bioinformatics/btz682 PMC7703786

[31] Lewis P, Ott M, Du J, et al. Pretrained language models for biomedical and clinical tasks: understanding and extending the state-of-the-art. In: Proceedings of the 3rd clinical natural language processing workshop; 2020. pp. 146–157.

[32] Li J, Sun Y, Johnson RJ, et al. Biocreative v cdr task corpus: a resource for chemical disease relation extraction. Database 2016. 10.1093/database/baw068 PMC4860626 27161011

[33] Lipscomb CE. Medical subject headings (mesh). Bull Med Libr Assoc. 2000;88(3):265. 10928714 PMC35238

[34] Liu P, Yuan W, Fu J, et al. Pre-train, prompt, and predict: A systematic survey of prompting methods in natural language processing. ACM Comput Surv. 2023;55(9):1–35.

[35] Luo Y, Yang K, Hong M, et al. Learning multi-view molecular representations with structured and unstructured knowledge. In: Proceedings of the 30th ACM SIGKDD conference on knowledge discovery and data mining. 2024. pp. 2082–2093.

[36] Maglott D, Ostell J, Pruitt KD, et al. Entrez gene: gene-centered information at ncbi. Nucleic acids research 39(suppl_1). 2010. pp. D52–D57. 10.1093/nar/gkq1237 PMC3013746 21115458

[37] McInnes BT, Tang J, Mahendran D, et al. Biobert-based deep learning and merged chemprot-drugprot for enhanced biomedical relation extraction. 2024. arXiv preprint arXiv:2405.18605

[38] Sänger M, Leser U. Large-scale entity representation learning for biomedical relationship extraction. Bioinformatics. 2021;37(2):236–42. 32726411 10.1093/bioinformatics/btaa674

[39] Sousa DF, Couto FM. K-ret: knowledgeable biomedical relation extraction system. Bioinformatics. 2023;39(4):btad174. 37018156 10.1093/bioinformatics/btad174 PMC10112952

[40] Sun Z, Deng ZH, Nie JY, et al. Rotate: knowledge graph embedding by relational rotation in complex space. In: International conference on learning representations; 2018.

[41] Tang X, Tran A, Tan J, et al. Mollm: a unified language model for integrating biomedical text with 2d and 3d molecular representations. Bioinformatics. 2024;40(1):i357–68. 38940177 10.1093/bioinformatics/btae260 PMC11256921

[42] UniProt Consortium and others. Uniprot: the universal protein knowledgebase in 2021. Nucleic Acids Res. 2021;49(D1):D480-9. 33237286 10.1093/nar/gkaa1100 PMC7778908

[43] Weber L, Sänger M, Garda S, et al. Chemical–protein relation extraction with ensembles of carefully tuned pretrained language models. Database 2022. 10.1093/database/baac098 PMC9674024 36399413

[44] Wei CH, Allot A, Leaman R, et al. Pubtator central: automated concept annotation for biomedical full text articles. Nucleic Acids Res. 2019;47(W1):W587–93. 31114887 10.1093/nar/gkz389 PMC6602571

[45] Wei CH, Allot A, Lai PT, et al. Pubtator 3.0: an ai-powered literature resource for unlocking biomedical knowledge. Nucleic Acids Res. 2024;52(1):540–6. 10.1093/nar/gkae235 PMC11223843 38572754

[46] Weininger D. Smiles, a chemical language and information system. 1. introduction to methodology and encoding rules. J Chem Inf Comput Sci. 1988;28(1):31–6.

[47] Wolf T, Debut L, Sanh V, et al. Huggingface’s transformers: state-of-the-art natural language processing. 2019. arXiv preprint arXiv:1910.03771.

[48] Yasunaga M, Leskovec J, Liang P. Linkbert: pretraining language models with document links. In: Proceedings of the 60th annual meeting of the association for computational linguistics. Association for computational linguistics. 2022. Vol. 1, pp. 8003–801.10.18653/V1/2022.ACL-LONG.551.

[49] Zhang D, Mohan S, Torkar M, et al. A distant supervision corpus for extracting biomedical relationships between chemicals, diseases and genes. In: Proceedings of the 13th language resources and evaluation conference. European Language Resources Association; 2022.

[50] Zheng Y, Guo Y, Luo Z, et al. A survey on document-level relation extraction: methods and applications. In: 3rd International conference on internet, education and information technology (IEIT 2023), Atlantis Press. 2023. pp. 1061–1071.

[51] Zhou D, Zhong D, He Y. Biomedical relation extraction: from binary to complex. Comput Math Methods Med. 2014;2014:1–18. 10.1155/2014/298473 PMC4156999 25214883

[52] Zhou W, Huang K, Ma T, et al. Document-level relation extraction with adaptive thresholding and localized context pooling. In: Proceedings of the AAAI conference on artificial intelligence. 2021. pp. 14612–14620.

